# Neuroprotective and Antioxidant Activity of Newly Synthesized N-Pyrrolyl Hydrazide-Hydrazones in Experimental Models of Neurotoxicity In Vitro and In Vivo

**DOI:** 10.3390/ijms27010370

**Published:** 2025-12-29

**Authors:** Martin Manov, Denitsa Stefanova, Magdalena Kondeva-Burdina, Yordan Yordanov, Martin Sharkov, Diana Tzankova, Emilio Mateev, Maya Georgieva, Georgi Popov, Vasil Manov, Maria Frosini, Massimo Valoti, Virginia Tzankova

**Affiliations:** 1 Department of Pharmacology, Pharmacotherapy and Toxicology, Faculty of Pharmacy, Medical University—Sofia, 1000 Sofia, Bulgaria; denitsa.stefanova@pharmfac.mu-sofia.bg (D.S.); mkondeva@pharmfac.mu-sofia.bg (M.K.-B.); yyordanov@pharmfac.mu-sofia.bg (Y.Y.); 2 Department of Pharmaceutical Chemistry, Faculty of Pharmacy, Medical University—Sofia, 1000 Sofia, Bulgaria; martin.sharkov1@abv.bg (M.S.); d.tsankova@pharmfac.mu-sofia.bg (D.T.); e.mateev@pharmfac.mu-sofia.bg (E.M.); mgeorgieva@pharmfac.mu-sofia.bg (M.G.); 3 Department of Internal Non-Communicable Diseases, Pathology and Pharmacology, Faculty of Veterinary Medicine, University of Forestry, 1111 Sofia, Bulgaria; gpopov@ltu.bg (G.P.); vmanov@ltu.bg (V.M.); 4 Department of Life Sciences, University of Siena, 53100 Siena, Italy; maria.frosini@unisi.it (M.F.); valoti@unisi.it (M.V.)

**Keywords:** N-pyrrolyl hydrazide-hydrazones, SH-SY5Y cells, 6-OHDA, MPP^+^ model, oxidative stress, rotenone-induced toxicity

## Abstract

Oxidative stress plays a central role in the pathogenesis of neurodegenerative disorders, including Parkinson’s disease. Therefore, compounds with antioxidant and neuroprotective properties represent promising candidates for therapeutic development. N-pyrrolyl hydrazide-hydrazones, a class of pyrrole-based derivatives, have shown promising potential due to their diverse biological activities, including monoamine oxidase-B (MAO-B) inhibition. This study investigated the neuroprotective properties of 10 N-pyrrolyl hydrazide-hydrazones using experimental in vitro and in vivo models of neurodegeneration. The compounds were tested on SH-SY5Y neuroblastoma cells subjected to oxidative stress induced by 6-hydroxydopamine (6-OHDA) and 1-methyl-4-phenylpyridinium (MPP^+^). A battery of in vitro and in vivo experimental methods was used, including cell viability assay, reactive oxygen species (ROS) production, and apoptosis evaluation by quantifying the sub-G0/G1 cell population. In vivo neuroprotective efficacy was further tested in a rotenone-induced Parkinsonism mouse model by analyzing oxidative biomarkers and brain histopathology. Compounds **2**, **4**, **5**, **6**, and **10** significantly preserved cell viability in the 6-OHDA-induced toxicity model, while no protection was observed in the MPP^+^ model. Particularly compound **2** reduced ROS levels and apoptosis in SH-SY5Y cells. In vivo, compound **2** demonstrated strong antioxidant activity by maintaining glutathione levels and reducing lipid peroxidation. Histological analysis confirmed its protective effect against rotenone-induced neuronal damage. These results suggest that N-pyrrolyl hydrazide-hydrazones, especially compound **2**, possess significant antioxidant and MAO-B inhibitory properties, supporting their potential as neuroprotective agents.

## 1. Introduction

Neurodegenerative diseases are characterized by a variety of pathophysiological mechanisms that result in progressive neuronal damage and dysfunction [1]. For instance. Parkinson’s disease (PD) involves the aggregation of α-synuclein and the formation of Lewy bodies, along with the death of dopaminergic neurons, particularly within the substantia nigra pars compacta (SNpc) [2]. The neurodegenerative process involves mitochondrial impairment and the excessive generation of reactive oxygen species (ROS), which further exacerbate neuronal injury [3]. Other neurodegenerative conditions display distinct hallmarks: multiple sclerosis (MS) is characterized by demyelination; amyotrophic lateral sclerosis (ALS) by extensive astrogliosis and astrocyte abnormalities; and Huntington’s disease (HD) by the accumulation of misfolded proteins [4]. Alzheimer’s disease (AD) is defined by the accumulation of β-amyloid (Aβ)-containing extracellular plaques and tau-containing intracellular neurofibrillary tangles, which lead to cognitive impairment [5].

At the molecular level, these processes are commonly associated with genetic mutations, dysregulation of the ubiquitin-proteasome-autophagy pathway, impaired bioenergetics, and deficits in neurotrophic signaling [6]. Conventional pharmacotherapy for these diseases—such as dopamine replacement (levodopa), monoamine oxidase-B (MAO-B) inhibitors (selegiline, rasagiline), and COMT (catechol-O-methyltransferase) inhibitors (entacapone, tolcapone) for PD—primarily provides symptomatic relief rather than neuroprotection or disease modification. The long-term efficacy of these therapies declines over time and is associated with significant adverse events [7].

Despite significant advances, current treatment strategies fail to halt or reverse the neurodegenerative process. Their limited bioavailability, poor blood-brain barrier (BBB) penetration, and non-selective systemic effects contribute to suboptimal outcomes. Furthermore, oxidative stress, mitochondrial dysfunction, and neuroinflammation remain largely unaddressed by conventional agents. Consequently, there is an increasing scientific focus on identifying novel small molecules capable of modulating multiple pathological pathways simultaneously—such as oxidative stress, mitochondrial impairment, and neuroinflammation—while exhibiting improved pharmacological safety and selectivity.

In recent years, pyrrole derivatives, which are nitrogen-containing heterocycles, have emerged as promising multifunctional neuroprotective agents due to their antioxidant, anti-inflammatory, and enzyme inhibitory activities [8]. The antioxidant, anti-inflammatory, and enzyme-inhibitory activities of the tested compounds may be explained by several mechanisms. The active N–H group in the molecules (where available) participates in free radical scavenging, while substituents on the ring appear to enhance stability and electron density. The appearance of a free OH group in some of the structures is also a prerequisite for enhanced radical scavenging effects, with hydroxylic groups being famous for their antioxidative properties in general. Additionally, the suppression of the compounds of key cytokines (TNF-α, IL-6) and inhibition of the NF-κB/NLRP3 inflammasome likely reduce inflammation. Together, these actions seem to mitigate oxidative damage and help preserve cellular and enzyme function, which may underlie the observed neuroprotective properties of the test compounds [9].

A series of pyrrolyl hydrazide and related azomethine derivatives with various pharmacophores were synthesized, demonstrating significant antioxidant and radical-scavenging activities comparable to standard antioxidants such as ascorbic acid and Trolox [10,11,12,13]. These compounds also exhibit MAO-B inhibitory activity, positioning them as dual-acting agents—combining antioxidant defense with modulation of dopamine metabolism [14,15,16]. The dual mechanism of action includes simultaneous ROS scavenging and MAO-B inhibition and suggests that these pyrrole-based azomethine compounds could mitigate both oxidative and enzymatic contributors to dopaminergic neuronal loss. Thus, they represent an innovative pharmacological strategy for the treatment of Parkinson’s disease and related neurodegenerative disorders.

To support this goal, the establishment of relevant in vitro models simulating disease-specific mechanisms is essential. These systems allow for the elucidation of toxicodynamic and pharmacodynamic relationships, facilitating rational drug design and screening [17]. Among the commonly used neurotoxic models are 6-hydroxydopamine (6-OHDA), MPTP (1-methyl-4-phenyl-1,2,3,6-tetrahydropyridine)/MPP^+^ (1-Methyl-1-PhenylPyridinium), and rotenone, which mimic Parkinsonian pathology through oxidative stress and mitochondrial dysfunction. 6-OHDA is a hydroxylated analogue of dopamine, which selectively enters dopaminergic neurons via monoamine transporters, where it generates ROS, inhibits antioxidant systems, and disrupts mitochondrial integrity [18]. MPTP crosses the BBB and is metabolized by astrocytic MAO-B into MPP^+^, which accumulates in dopaminergic neurons and inhibits mitochondrial complex I, triggering ROS formation and apoptosis [19,20]. Similarly, rotenone—a natural pesticide—induces dopaminergic degeneration, α-synuclein aggregation, and behavioral deficits in rodents, closely mimicking idiopathic Parkinson’s disease [21].

Considering the accumulated evidence on the multifactorial mechanisms of neurodegeneration and the potential of pyrrole-based azomethine derivatives, the aim of the present study is to evaluate the neuroprotective potential of selected pyrrole-based azomethine compounds, considering their established antioxidant and MAO-B inhibitory activities. A comprehensive battery of in vitro and in vivo models of neurotoxicity was applied, including 6-hydroxydopamine (6-OHDA), MPP^+^, and rotenone-induced toxicity. The study aims to determine the ability of these compounds to mitigate key pathological processes, such as oxidative stress, mitochondrial dysfunction, and dopaminergic neuronal loss, thereby providing a promising and mechanistically grounded therapeutic approach for neurodegenerative diseases.

## 2. Results

### 2.1. Selection of the Targeted Pyrrole-Based Azomethine Derivatives

Classical reactions for synthesis of the targeted molecules were applied, as depicted in [22] for synthesis of compounds **5**, **6**, and **7**; [23] for synthesis of **8**, **9**, and **10**; [24] for synthesis of **4**; [25] for synthesis of **1**; and [26] for synthesis of **2** and **3**. The corresponding preliminary evaluation of radical scavenging effects of the target derivatives was evaluated, based on the DPPH and ABTS in vitro tests and from the obtained analysis, presented in the articles, published previously [11,24,27]. Additional evaluations on possible MAO-B inhibitory activity [15,26], along with combined MAO-B inhibition and antioxidant effects, were also performed [28]. The most promising antioxidants, MAO-B inhibitors, and dual radical-scavenging/MAO-B-inhibiting compounds identified in our previous studies were extracted and selected and are listed in the current paper under their designated codes (Table 1).

The selection of the proposed derivatives was based on preliminary analysis discussing the identified antioxidant, MAO-B inhibition, and combined radical scavenging/MAO-B inhibiting activity of a series of pyrrole-based azomethine compounds synthesized in our laboratory.

### 2.2. Safety Evaluation of the New N-Pyrrolyl Hydrazide-Hydrazones

**Figure 1:**
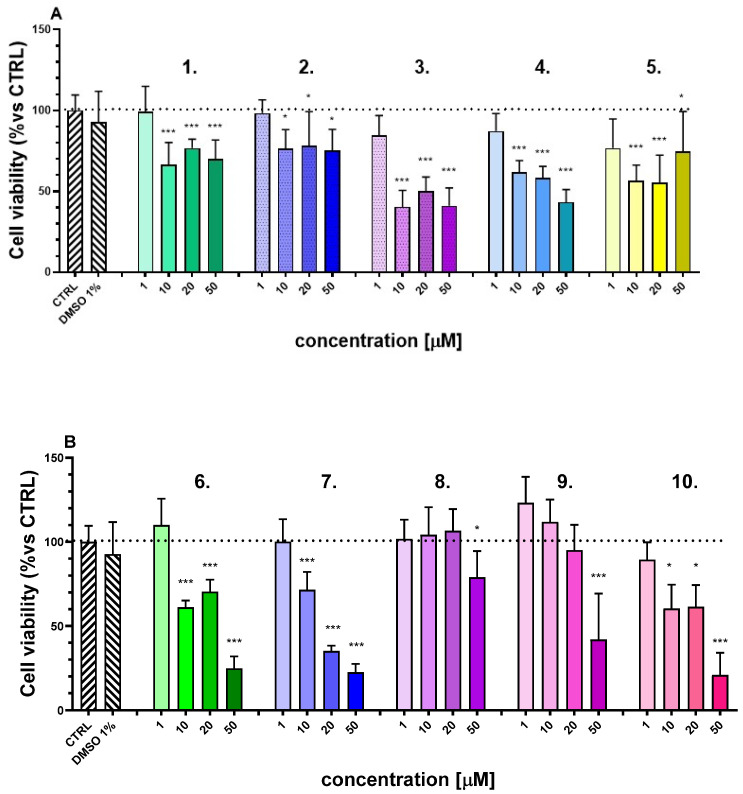
In vitro cytotoxicity assessment in human neuroblastoma cell line SH-SY5Y, treated with the test compounds at concentrations of 1, 10, 20, and 50 μM (**A**) compounds **1**–**5**, (**B**) compounds **6**–**10**. Data are presented as mean ± SEM. Statistical analysis was performed using ANOVA with Dunnett’s post-test * *p* < 0.05, *** *p* < 0.001. Results from three independent experiments are presented.

To determine non-toxic concentration ranges for newly synthesized compounds, SH-SY5Y cells were exposed to a series of increasing compound concentrations (1, 10, 20, and 50 µM) (Figure 1). After a 24 h incubation period, cell viability was evaluated using the MTT assay, a colorimetric technique that measures mitochondrial metabolic activity.

Following the in vitro toxicity evaluation, the specific non-toxic concentrations of the compounds, i.e., the highest concentration that did not produce a significant reduction in cell viability after 24 h of treatment, were identified and selected for further experimental investigation (Table 2). The concentration of 1 μM was chosen for the compounds **1**–**7** and compound **10** for the next in vitro neuroprotection experiments to avoid cytotoxic effects that could interfere with the interpretation of the protective outcomes. Our preliminary toxicity testing demonstrated no toxic effects for compounds **8** and **9** even at higher concentrations (20 μM); thus, we considered it appropriate to use these higher concentrations to obtain the maximal achievable neuroprotection for both compounds.

### 2.3. Neuroprotective Effects of Newly Synthesized N-Pyrrolyl Hydrazide-Hydrazones in a Model of 6-OHDA-Induced Neurotoxicity

**Figure 2:**
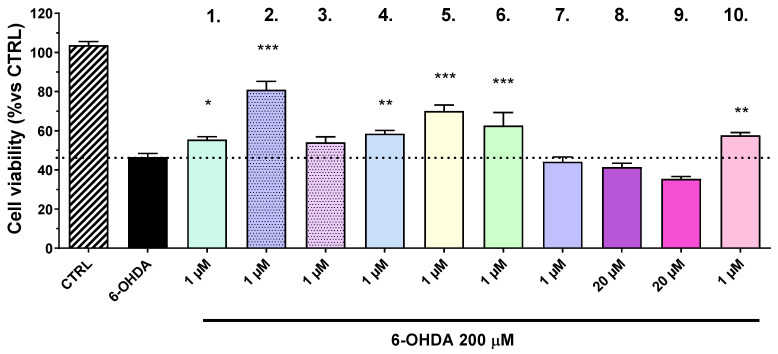
Neuroprotective effects of the test compounds (**1**–**10**) in a model of neuronal damage with 6-OHDA in the human neuroblastoma cell line SH-SY5Y. Concentrations used are selected according to the results presented in Figure 1. Data are presented as mean ± SEM. Statistical analysis was performed using ANOVA with Dunnett’s post-test; * *p* < 0.05, ** *p* < 0.01, and *** *p* < 0.001.

The results demonstrated a statistically significant protective effect for the tested compounds, respectively by **2** (31%), **5** (20%), and **6** (12.6%) (*p* < 0.001), as well as for compounds **4** (8.5%) and **10** (7.6%) (*p* < 0.01) (Figure 2).

### 2.4. Neuroprotective Effects of Newly Synthesized N-Pyrrolyl Hydrazide-Hydrazones in a Model of MPP+-Induced Neurotoxicity

**Figure 3:**
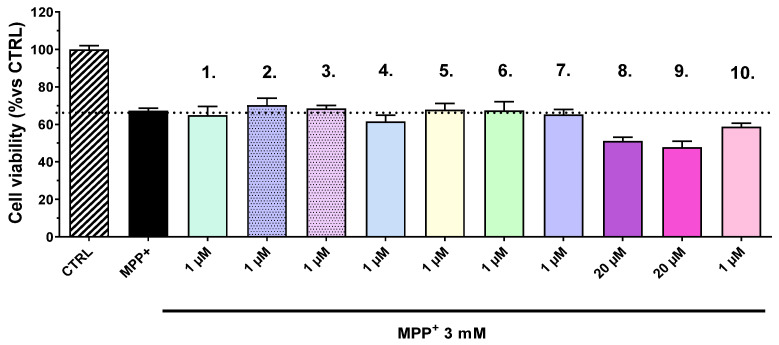
Effects of the test compounds in the model of neuronal degeneration induced by MPP+ on human neuroblastoma cell line SH-SY5Y. Data are presented as mean ± SEM. Statistical analysis was performed using ANOVA with Dunnett’s post-test.

Cell pre-treatment with the compounds was conducted for 1 h, and then a 1.5 mM solution of MPP^+^ was added to each well (Figure 3). In this in vitro model of neuronal injury, none of the tested compounds exhibited neuroprotective effects.

### 2.5. Effects of the Tested Compounds (1 µM) on the Activity of Human Recombinant MAO-A and MAO-B Enzymes (hMAO-A and hMAO-B)

**Figure 4:**
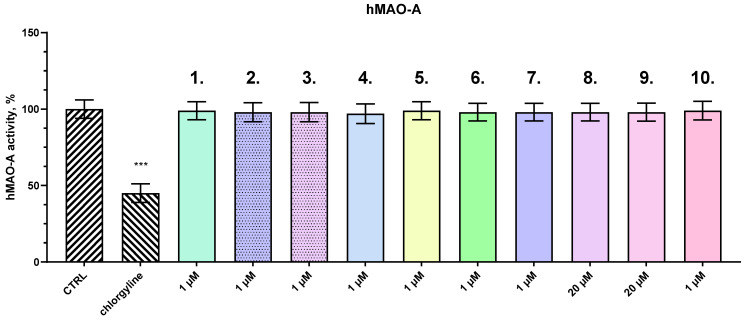
Effects of compounds **1**, **2**, **3**, **4**, **5**, **6**, **7**, **8**, **9**, and **10**, and chlorgyline (concentration 1 µM) on the activity (%) of human recombinant MAO-A enzyme (hMAO-A). Data are presented as mean ± SD. Statistical analysis was performed using ANOVA with Dunnett’s post-test; *** *p* < 0.001.

To evaluate the potential inhibitory effects of the tested compounds on human monoamine oxidase A (hMAO-A), an in vitro enzyme inhibition assay was conducted. In our previous studies, a concentration of 1 µM was used to determine the inhibitory effect on human recombinant MAO-A and MAO-B enzymes [15]. The compounds were incubated with purified hMAO-A enzyme for a period of 2 h under standard assay conditions. Enzyme activity was measured spectrophotometrically and compared to a control sample containing hMAO-A alone, which was set as the baseline for 100% activity. As shown in Figure 4, none of the tested compounds demonstrated a statistically significant reduction in enzymatic activity relative to the control, indicating a lack of meaningful inhibitory effect on hMAO-A under the experimental conditions used. In contrast, chlorgyline—a well-established, irreversible MAO-A inhibitor—served as the positive control and significantly decreased enzyme activity by approximately 55%, thereby validating the sensitivity and accuracy of the assay. These findings suggest that the tested compounds do not interact with or inhibit hMAO-A to a relevant extent and therefore are unlikely to exert monoaminergic effects via this enzymatic pathway, at least at the tested concentration.

**Figure 5:**
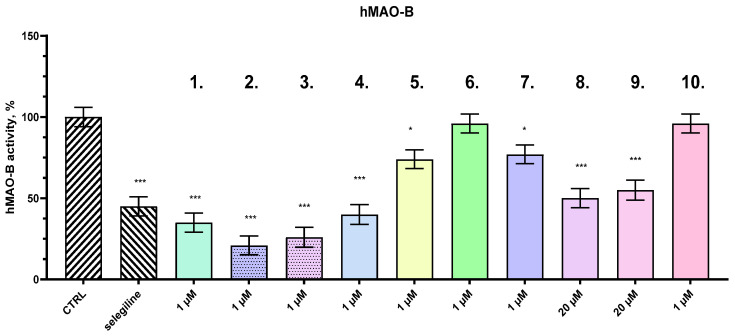
Effects of compounds **1**, **2**, **3**, **4**, **5**, **6**, **7**, **8**, **9**, and **10**, and selegiline (concentration 1 µM) on the activity (%) of human recombinant MAO-B enzyme (hMAO-B). Data are presented as mean ± SD. Statistical analysis was performed using ANOVA with Dunnett’s post-test; * *p* < 0.05, *** *p* < 0.001.

All the tested compounds and selegiline were evaluated for possible MAO-B inhibitory activity. The compounds were incubated with purified hMAO-B enzyme for 2 h, and enzyme activity was quantified using a standard spectrophotometric assay. Several of the tested compounds demonstrated potent and significant inhibitory effects on hMAO-B activity, with some compounds exhibiting greater inhibition than selegiline, a well-known, selective inhibitor of hMAO-B. The in vitro concentration of 1 µM is equal to the plasma concentration (0.89 µM) during the in vivo experiment. The results, shown in Figure 5, indicate that a subset of the compounds achieved notable reductions in hMAO-B activity, with inhibition levels surpassing that of selegiline, which inhibited the enzyme by 55%.

Among the most effective compounds, **2** demonstrated the highest inhibitory effect, reducing hMAO-B activity by 79%, followed by **3** (74%), **1** (65%), and **4** (60%). These compounds displayed superior inhibition compared to selegiline, suggesting they may offer enhanced efficacy for therapeutic interventions targeting diseases related to excessive monoamine oxidase B activity, such as Parkinson’s disease. Interestingly, compounds **6** and **10** showed no significant inhibitory effects on hMAO-B, indicating a lack of interaction with the enzyme under the tested conditions.

### 2.6. Intracellular ROS Generation

**Figure 6:**
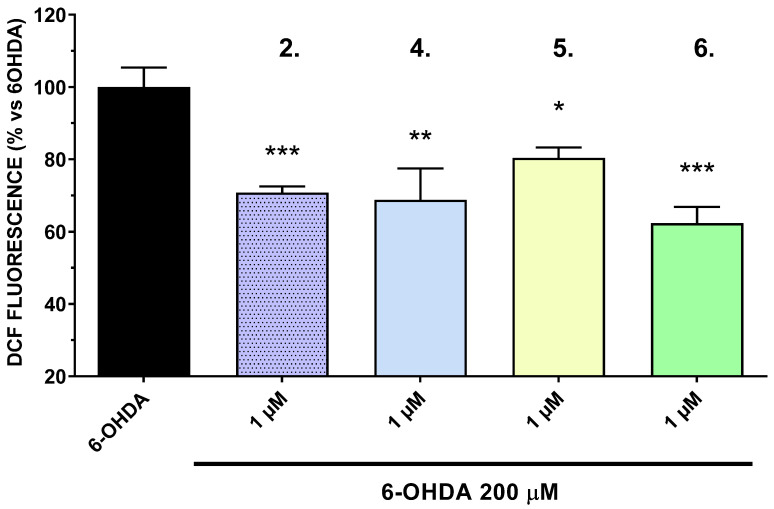
Effects of the test compounds at concentration 1 μM on intracellular generation of ROS, measured by the DCFH-DA method and compared to the effect of 6-OHDA 200 µM. Data are presented as mean ± SEM. Statistical analysis was performed using ANOVA with Dunnett’s post-test. * *p* < 0.05, ** *p* < 0.01; *** *p* < 0.001. Data were normalized to the fluorescence measured in the 6-OHDA–treated control group.

The neurotoxic effects of 6-OHDA are primarily attributed to its ability to generate ROS, leading to oxidative stress and subsequent neuronal damage. By attenuating ROS production, the tested compounds may offer neuroprotective benefits, potentially through mechanisms involving the scavenging of free radicals or the enhancement of endogenous antioxidant defenses. In this study, the intracellular production of reactive oxygen species (ROS) was assessed in the SH-SY5Y cell line, focusing on the most active compounds (**2**, **4**, **5**, and **6**) identified in the 6-hydroxydopamine (6-OHDA) model. 6-OHDA served as the positive control, with its ROS production activity normalized to 100%. At a concentration of 1 µM (based on the results presented in Figure 1), compounds **2** and **6** significantly reduced ROS production (*p* < 0.001), with reductions to 70.7% and 62.3%, respectively. Compound **4** also demonstrated a significant reduction in ROS levels (*p* < 0.01) (Figure 6). These findings suggest that these compounds possess potential antioxidant properties, capable of mitigating oxidative stress induced by 6-OHDA.

Further investigations are warranted to elucidate the precise pathways through which these compounds exert their effects and to assess their potential therapeutic applications in neurodegenerative conditions characterized by oxidative stress.

### 2.7. Analysis of the Influence on the Cell Cycle of the Tested Compounds

**Figure 7:**
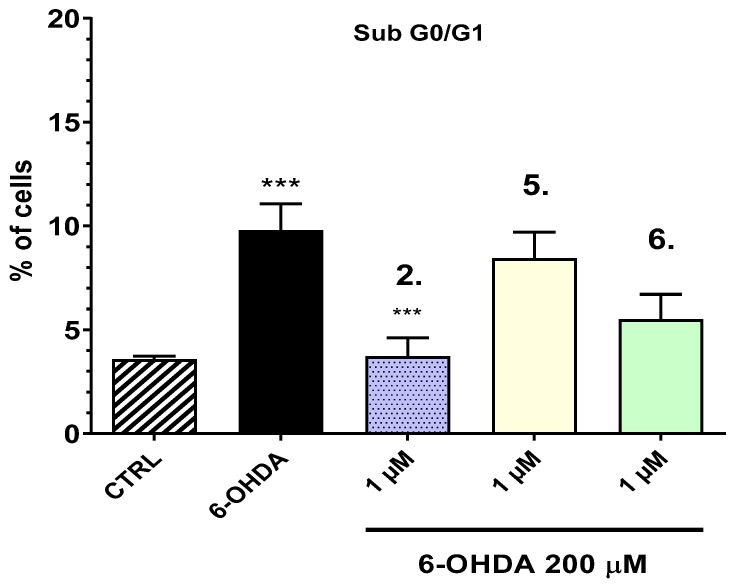
Effects of compounds **2**, **5**, and **6** on 6OHDA-induced changes in the sub-G0/G1 population of SH-SY5Y determined by flow cytometry after propidium iodide staining. The graph displays the percentage of cells undergoing apoptosis (sub-G0/G1 phase), determined by flow cytometry after propidium iodide staining. *** *p* < 0.001.

Compounds **2**, **5**, and **6** showed the highest potency to preserve the cell viability in the model of neurotoxicity with 6-OHDA and were selected for this assay. To further investigate the mechanisms underlying 6-hydroxydopamine (6-OHDA)-induced injury and the potential protective effects of the tested compounds (**2**, **5**, and **6**), the sub-G0/G1 cell population was quantified using flow cytometry, as it corresponds to apoptotic cells characterized by fragmented DNA (Figure 7).

The cells treated with 6-OHDA exhibited a significant increase in the sub-G0/G1 population, with 9.785% of cells undergoing apoptosis, confirming the pro-apoptotic effect of 6-OHDA. The increase in sub-G0/G1 is accompanied by a decrease in cells in G0/G1, typical of apoptosis, while the percentage of cells in the other phases of the cycle remains unchanged. However, compound **2** (1 µM) markedly reduced the percentage of apoptotic cells to 3.73%, indicating a statistically significant protective effect compared to the 6-OHDA control (Additional data are provided in Supplementary Materials, Figure S1). The observed reduction suggests that compound **2** may confer neuroprotective properties by mitigating 6-OHDA-induced apoptosis. These findings underscore the potential of compound **2** as a candidate for further investigation in the context of neurodegenerative diseases characterized by oxidative stress and apoptotic cell death.

### 2.8. In Vivo Evaluation of the Compounds in a Rotenone-Induced Parkinsonism Mouse Model

In the rotenone-induced Parkinsonism mouse model, rotenone primarily induces toxicity by inhibiting mitochondrial complex I [30], leading to impaired cellular respiration and increased ROS production. This results in oxidative stress, mitochondrial dysfunction, and dopaminergic neuron degeneration, mimicking the neurodegenerative processes seen in Parkinson’s disease.

The in vivo neuroprotective and toxicological effects of the most active test compounds—**2**, **4**, and **5**—were assessed using a well-established rotenone-induced Parkinsonism model in mice. When administered individually, these compounds demonstrated relatively low neurotoxicity, as measured by their effects on oxidative stress biomarkers in the brain.

Specifically, compound **4** caused a moderate depletion of glutathione (GSH) levels by approximately 20% and a corresponding increase in malondialdehyde (MDA) production by 56%, compared to the untreated control group (healthy mice). Compound **5** induced a more pronounced reduction in GSH levels (30%) along with a 33% elevation in MDA levels. Likewise, compound **2** led to a 15% decrease in GSH levels and a 67% rise in MDA concentrations. These results indicate a mild to moderate oxidative impact, but significantly less severe than the effect of the neurotoxic agent rotenone.

**Figure 8:**
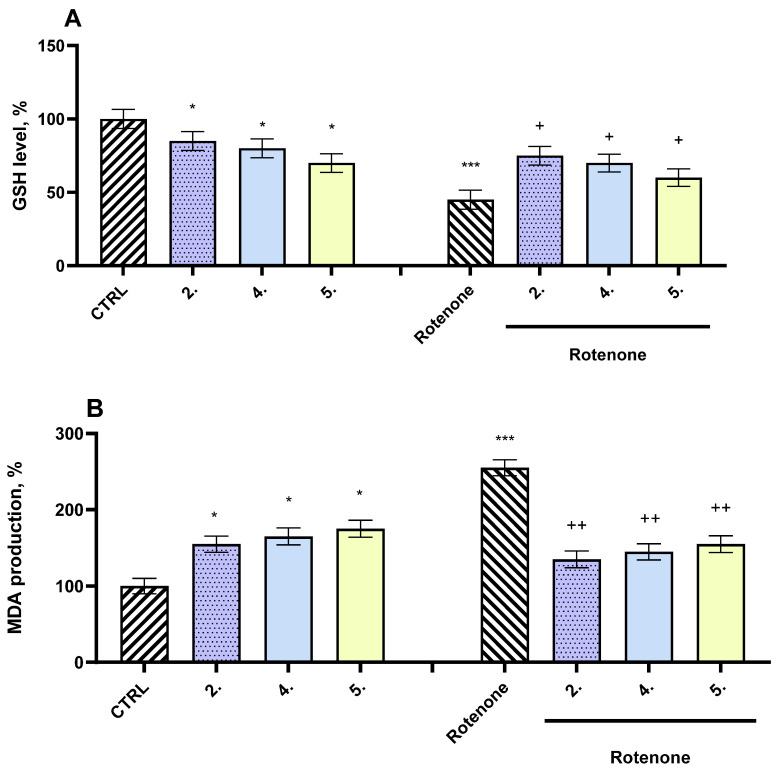
In vivo effects of compounds **2**, **4**, and **5**, administered alone and co-administered with rotenone, on the brain homogenate GSH level (**A**) and MDA production (**B**). * *p* < 0.05 vs. control (pure mice); *** *p* < 0.001 vs. control (pure mice); + *p* < 0.05 vs. control (pure rotenone); ++ *p* < 0.01 vs. control (pure rotenone).

By comparison, administration of rotenone alone induced substantial toxicity. It markedly reduced brain GSH levels by 55% and caused a dramatic 155% increase in MDA levels relative to the control group (Figure 8). These findings confirm the strong oxidative stress and lipid peroxidation associated with rotenone exposure, which is consistent with its known mechanism of mitochondrial dysfunction and ROS overproduction.

Overall, while the test compounds showed some oxidative effects, they were significantly less toxic than rotenone, suggesting potential neuroprotective properties.

When co-administered with rotenone, all tested compounds (**2**, **4**, and **5**) exhibited significant neuroprotective effects in the mouse model. Compound **2** showed the strongest effect, preserving GSH levels by 67% and reducing MDA by 47%, all compared to the rotenone-only group (Figure 8). Compound **4** preserved GSH levels by 56% and reduced MDA production by 43%. Compound **5** maintained GSH levels by 33% and decreased MDA by 39%.

The observed neuroprotective effects of compounds **2**, **4**, and **5** may be associated with their ability to inhibit MAO-B, a key mitochondrial enzyme responsible for the oxidative deamination of dopamine and the metabolism of neurotoxins such as rotenone. By inhibiting MAO-B, these compounds may reduce the production of hydrogen peroxide and other ROS, thereby mitigating oxidative stress and neuronal damage commonly observed in rotenone-induced Parkinsonism.

### 2.9. Histopathological Analysis

**Figure 9:**
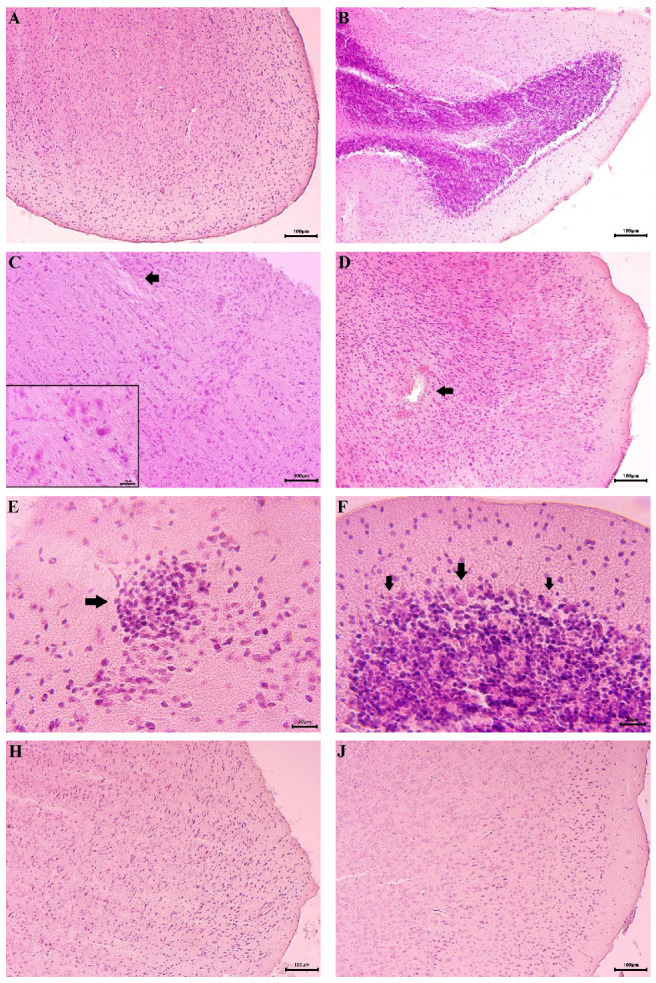

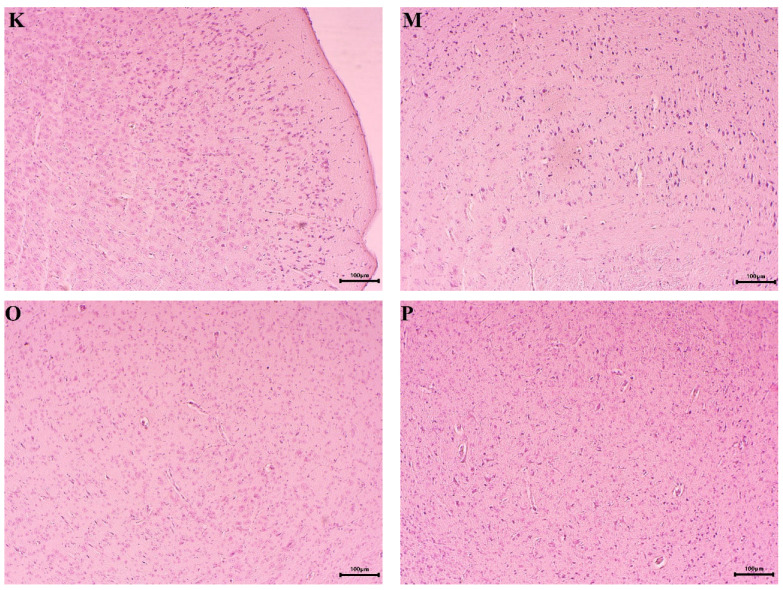
Histological examination of mouse brains. (**A**) control group of animals: normal histoarchitectonics of the cerebral cortex; (**B**) control group of animals: normal histological structure of the cortex of cerebellum; (**C**) rotenone-treated animal: loss of intercellular space integrity (arrow) and pyknotic changes in ganglion cells (high magnification); (**D**) rotenone-treated animal: degenerative-necrotic area with hemorrhage in brain tissue (arrow); (**E**) rotenone-treated animal: glial nodule in brain parenchyma (arrow); (**F**) cerebellum of rotenone-treated animal: pyknotic changes in Purkinje cells (arrows); (**H**) animal treated with **2** alone: normal brain structure; (**J**) animal treated with **4** alone: normal brain structure; (**K**) animal treated alone with **5**: normal brain structure; (**M**) animal treated with combination of **2** with rotenone: unaltered brain structure; (**O**) animal treated with combination of **4** with rotenone: unaltered brain structure; (**P**) animal treated with combination of **5** with rotenone: unaltered brain structure.

The histological structure of the mouse brains, particularly the cerebellar cortex and cerebellum, was examined under a microscope. Light microscopy revealed no morphological alterations in the ganglion or glial cells of the brain structures in the control group. The cerebral cortex displayed a normal layered organization, with indistinct boundaries between individual layers (Figure 9A). Cortical neurons exhibited a typical histological appearance, featuring oval nuclei and pale cytoplasm. Astrocytes were visible in the neuropil without signs of pathological changes, and the blood vessels maintained intact perivascular spaces. The cerebellum also showed no detectable abnormalities; its cortical zone presented a well-defined laminar structure, including a clearly delineated outer molecular layer, a middle Purkinje cell layer, and an inner granule cell layer (Figure 9B).

In contrast, the brains of mice treated with rotenone showed distinct degenerative and necrotic changes in the cerebral cortex. These included shrunken neurons with intensely eosinophilic, dark-stained cytoplasm and pyknotic nuclei. Disruption of tissue integrity and neuropil vacuolization were also observed (Figure 9C). Additionally, hemorrhagic areas were noted in parts of the brain parenchyma (Figure 9D). In the cerebellar cortex, glial nodules were present, primarily localized in the peripheral zones (Figure 9E). The most pronounced pathological changes were found in the Purkinje cell layer, where cells were deformed, shrunken, and had lost their characteristic pyriform shape. These cells displayed irregular contours, dark-stained cytoplasm, and poorly defined nucleoli (Figure 9F). Furthermore, the typical linear arrangement of Purkinje cells was disrupted, leading to marked disorganization in affected regions.

In mice treated only with compounds **2**, **4**, and **5**, no microscopically visible alterations were observed, and the histological architecture remained comparable to that of the control group (Figure 9H,J,K).

Light microscopy of brain sections from mice treated with a combination of rotenone and compounds **2**, **4**, and **5** revealed preserved histoarchitecture (Figure 9M,O,P). The ganglion and glial cells appeared intact, with no visible pathological changes, although occasional isolated glial nodules were observed in specific brain areas.

## 3. Discussion

Some preliminary studies on the in vitro radical scavenging properties of the selected molecules were used for screening and preselection of promising compounds for additional studies in a panel of various tests, aiming to indicate a possible mechanism of antioxidant action, which will point to further development of analogues of this chemical class. The evaluation of in vivo activity of the selected molecules is another contribution of the current study. The observations made based on the obtained experimental data defined that the introduction of the unsubstituted N-H group from the Tyr residue in the bridge may be considered the reason for the appearance of increased antioxidant capacity compared to representatives not containing this residue. On the other hand, the introduction of the resonance-active EDG (electron-donating group) OH group in the carbonyl fragment is believed to also add to the antioxidant capacity. The introduction of the electron-withdrawing –NO_2_ group in the carbonyl fragment does not affect the antioxidant properties remarkably. The same observations are found also for the substituted indole residue.

The results also defined that the availability of a free hydrazide –NH-NH_2_ group is a prerequisite for the identification of good radical scavenging properties as established in our finding as mentioned in [26,31].

As a result of the conclusions drawn from previous experiments, the structures presented in Table 1 were subjected to additional evaluations in appropriate in vitro and in vivo models, where the finding extracted compound **2** as the most promising. We believe this result is based on the combination of the available free Tyr residue in the bridge and additional phenyl-linked –OH EDG, related to antioxidant capacity overlay.

This study provides comprehensive insight into the biological activity, safety profile, and neuroprotective potential of newly synthesized N-pyrrolyl hydrazide-hydrazone derivatives using both in vitro and in vivo models relevant to PD. Our findings highlight several promising compounds, most notably **2**, **4**, and **5**, with significant protective activity against neurotoxin-induced cellular and tissue damage. The data collectively support the hypothesis that these compounds exert neuroprotection via multiple mechanisms, including attenuation of oxidative stress, inhibition of MAO-B, and anti-apoptotic effects. The initial cytotoxicity screening using SH-SY5Y human neuroblastoma cells identified the non-toxic concentration ranges for each compound, with most exhibiting low cytotoxicity even at higher concentrations (up to 50 µM). This is an essential prerequisite for further pharmacological evaluation, particularly when studying neuroprotective effects that could be confounded by intrinsic cytotoxicity. Assessment of mitochondrial metabolic activity via the MTT assay at the 24 h time point provided a reliable indicator of cell viability. The selected concentrations used in subsequent assays ensured that any observed biological effects were not secondary to general toxicity but instead reflective of specific pharmacological action.

In a subsequent series of experiments, we evaluated the effects of the compound in an in vitro model of 6-OHDA-induced neurotoxicity. The 6-OHDA model is a well-established in vitro model for Parkinsonian neurodegeneration, characterized primarily by the generation of excessive ROS and selective dopaminergic neuron loss [32]. Our results showed that compounds **2**, **5**, and **6** significantly increased cell viability in the presence of 6-OHDA, suggesting they can mitigate its cytotoxic effects. Compounds **4** and **10** also demonstrated moderate but statistically significant protective activities. Importantly, ROS quantification revealed that compounds **2** and **6** significantly reduced intracellular oxidative stress at low concentrations (1 µM). These antioxidant effects are central to the observed neuroprotection, as ROS are implicated in mitochondrial dysfunction, lipid peroxidation, and DNA fragmentation, all of which contribute to dopaminergic neuronal death in PD [33]. Further evidence supporting the neuroprotective capacity of these compounds was obtained via flow cytometric analysis of apoptosis. Treatment with compound **2** reduced the percentage of apoptotic cells (sub-G0/G1 population) from 9.79% (6-OHDA group) to 3.73%. This finding indicates that **2**, not only protects against oxidative stress but may also actively suppress apoptosis—likely through inhibition of pro-apoptotic signaling cascades or enhancement of cellular survival pathways such as PI3K/Akt or Bcl-2 expression. These dual actions of reducing ROS and apoptosis position compound **2** as a particularly compelling neuroprotective candidate.

The MPP^+^ model closely replicates the dopaminergic neuronal degeneration observed in PD. MPP^+^, the active metabolite of MPTP, selectively accumulates in dopaminergic neurons via the dopamine transporter (DAT) [34]. Within neurons, MPP^+^ localizes to mitochondria, where it inhibits complex I of the electron transport chain, leading to ATP depletion, increased oxidative stress, and ultimately, neuronal cell death. Interestingly, none of the tested compounds exhibited neuroprotective effects in the MPP^+^ model, a selective mitochondrial complex I inhibitor that replicates the mitochondrial dysfunction seen in PD [35]. This lack of efficacy may reflect the specific mechanistic limitations of the compounds, which seem more effective in combating oxidative stress than direct mitochondrial impairment. Whereas 6-OHDA acts by generating ROS both enzymatically and non-enzymatically [36], MPP^+^ exerts toxicity through inhibition of the mitochondrial electron transport chain, leading to ATP depletion and a cascade of secondary oxidative insults [37]. The inability of the tested compounds to protect cells in this model suggests that while they may possess strong antioxidant and anti-apoptotic properties, they might lack the ability to restore mitochondrial bioenergetics or protect complex I function directly. This distinction is crucial for understanding the scope of their therapeutic potential and highlights the need for combination therapies or compound optimization targeting broader neurodegenerative mechanisms. Nevertheless, considering the safety margin (up to 50 µM), we cannot exclude that increasing the concentrations would also lead to neuroprotective effects in the MPP^+^ model.

Furthermore, a particularly noteworthy finding from this study is the selective inhibition of MAO-B by several compounds, with no significant activity against MAO-A. The specificity of action is therapeutically desirable, as selective MAO-B inhibition increases dopamine availability in the brain while minimizing adverse cardiovascular effects associated with MAO-A inhibition. Compound **2** once again emerged as the most potent MAO-B inhibitor, as it reduces enzyme activity by 79%, outperforming the well-known MAO-B inhibitor selegiline at the concentration of 1 μM (55%).

This MAO-B inhibition is relevant in the context of PD not only for symptom management via increased dopamine availability but also for neuroprotection [38]. MAO-B metabolizes dopamine into hydrogen peroxide, a source of oxidative stress. Its inhibition reduces ROS production, thereby mitigating oxidative damage—a finding consistent with the observed reductions in ROS and MDA levels in both in vitro and in vivo settings [39].

The variation in inhibitory potency among the compounds suggests that structural features may play a critical role in their interaction with hMAO-B. Further investigation is required to understand the precise mechanisms underlying the observed inhibition and to evaluate the potential of these compounds as therapeutic agents. These findings open avenues for the development of more effective MAO-B inhibitors with potentially improved pharmacological profiles for neurodegenerative disorders.

The in vivo experiments using the rotenone-induced Parkinsonism mouse model further validated the neuroprotective potential of compounds **2**, **4**, and **5.** Rotenone, a potent mitochondrial complex I inhibitor, induces dopaminergic neuronal death by increasing ROS and lipid peroxidation, mimicking many aspects of PD pathophysiology [40].

We observed a strong in vivo antioxidant effect in our experiments, which is consistent with our in vitro findings. Together, these results suggest that the compound has a robust neuroprotective mechanism. Biochemical assays revealed that co-administration of the compounds with rotenone significantly preserved GSH levels and reduced MDA, a marker of lipid peroxidation [41]. These changes were most pronounced for compound **2**, which preserved GSH by 67% and reduced MDA by 47% relative to the rotenone-only group. This strong in vivo antioxidant effect is consistent with the in vitro data and highlights the compound’s robust neuroprotective mechanism.

Furthermore, histopathological analysis corroborated the biochemical findings. While rotenone-treated brains exhibited severe neuronal damage, including cortical necrosis, Purkinje cell degeneration, and glial activation, co-treatment with the test compounds preserved normal tissue architecture. Only minor glial nodules were observed in compound-treated groups, and the characteristic laminar structure of the cerebellar cortex remained intact. This morphological preservation aligns with the observed biochemical neuroprotection and further supports the therapeutic potential of these agents.

## 4. Materials and Methods

### 4.1. Cell Line

Neuroblastoma SH-SY5Y cells (Sigma-Aldrich, Merck, Darmstadt, Germany, passage 6–15), authenticated by genetic profiling (LGC Standards S.r.L., Milan, Italy), were cultured in Dulbecco’s Modified Eagle’s Medium (DMEM) low glucose, supplemented with 10% heat-inactivated fetal bovine serum (FBS), 1% L-glutamine, and 1% Penicillin/Streptomycin (100 U/mL and 100 µg/mL). The cells were maintained in a humidified incubator at 37 °C with 5% CO_2_. The growth medium was refreshed every two days, and the cells were subcultured upon reaching 80–90% confluence. All solutions used for cell culture were preheated to 37 °C before use.

### 4.2. Cell Viability Assay

Cell viability was assessed using the MTT assay (3-(4,5-dimethylthiazol-2-yl)-2,5-diphenyltetrazolium bromide) in human neuroblastoma SH-SY5Y cells. After all treatments, 200 µL of MTT solution (0.5 mg/mL in culture medium) was added to each well, and the plates were incubated for 3 h at 37 °C. Following incubation, the supernatant was aspirated, and 200 µL of DMSO was added to each well to extract and solubilize the formazan crystals. Absorbance was measured using a plate reader at a wavelength of 540 nm.

### 4.3. Selection of the Targeted Pyrrole Azomethines

Thirteen pyrrole-based hydrazide-hydrazones were selected for in vitro and in vivo evaluation of their neurotoxic and neuroprotective effects on different well-established models. The selection was based on previous investigations of the performed radical scavenging activity and inhibition of MAO-B effects.

### 4.4. 6-OHDA-Induced Model of Neurotoxicity in SH-SY5Y Cell Line

Cells were seeded in a 96-well plate at a density of 1.5 × 10^4^ cells per well and allowed to attach overnight. After 24 h, the cells were pre-treated for 1 h with different concentrations of the compounds and then 200 μM 6-OHDA for 3 h, plus compounds in concentrations stated in Table 2. The treatment in the control sample with 200 µM 6-OHDA was selected as it reduced cell viability to approximately 50%. 6-OHDA was prepared immediately before use by dissolving the powder in 5 μM ascorbic acid solution previously gassed with nitrogen for 30 min. Due to the photosensitive and chemically unstable nature of 6-OHDA, the treatment was conducted in darkness and as quickly as possible. Stock solutions of tested compounds were prepared in DMSO (1 × 10^−1^ M or 1 × 10^−2^ M) and diluted to the desired final concentration with PBS immediately before use. Final DMSO concentration in the samples was always lower than 0.01%, and it did not affect investigated parameters.

For each well, 20 µL of 2000 μM 6-OHDA solution and 20 µL of compound solution in 10 times higher concentration, indicated in Table 3, were added to 160 µL of medium. For control samples, 20 µL of 10× ascorbic acid (AA) solution was added to 180 µL of medium (final concentration of AA 0.001%). The control sample did not affect the cell viability in the test for cytotoxicity. The solutions were prepared in situ in the wells following a 1 h pre-treatment with the compounds.

### 4.5. MPP^+^-Induced Model of Neurotoxicity

Cells were seeded in a 96-well plate at a density of 1.5 × 10^4^ cells per well and allowed to attach overnight. After 24 h, the cells were pre-treated for 1 h with concentrations of the compounds, as stated in Table 2 and then 1.5 mM MPP^+^ for 24 h plus compounds in the same concentrations. The treatment in the control sample with 1.5 mM MPP^+^ was selected as it reduced cell viability to approximately 65%. MPP^+^ stock solution was freshly prepared before each treatment by dissolving the powder in cell culture medium at a concentration of 10 mM [42].

### 4.6. Determination of Human Recombinant MAO-A/B Enzyme Activity

The activity of recombinant human MAO-A/B was determined fluorimetrically using tyramine hydrochloride as the substrate. The activity was assessed by detecting H_2_O_2_ production, which was measured via its reaction with horseradish peroxidase, using N-acetyl-3,7-dihydroxyphenoxazine (Amplex Red) as the fluorescent reporter [43].

Working solutions of the test substances, reagents, and human recombinant MAO-A/B enzyme (hMAO-A/B) were prepared in reaction buffer according to the manufacturer’s instructions. Control samples included a pure working solution of MAO-A/B in reaction buffer, a working solution of MAO-A/B containing hydrogen peroxide, and a pure reaction buffer. Test substances were applied at a final concentration of 1 µM. The substances, along with hMAO-A/B, were added to a 96-well plate (8 replicates per substance), and the plate was incubated in the dark at 37 °C for 30 min. Fluorometric readings were conducted using a Synergy 2 Microplate Reader (BioTek Instruments, Inc., Highland Park, Winooski, VT, USA), measuring at two wavelengths: 570 nm and 690 nm.

### 4.7. Intracellular Generation of ROS

For compounds **2**, **4**, **5**, and **6**, the generation of ROS in living cells was assessed as described previously [44]. ROS generation was assessed in cells rinsed with PBS and loaded with 10 μM 2′,7′-dichlorofluorescin diacetate (DCFDA) for 10 min at 37 °C, then washed, centrifuged at 13,000× *g* for 5 min, and resuspended in 0.7 mL of PBS. The intracellular fluorescence (504 nm excitation, 529 nm emission, Fluoroskan Ascent fluorimeter, Thermo Labsystems, Helsinki, Finland) was normalized to mg of cellular protein of the samples and expressed as a percent of untreated-, control cells. Appropriate controls with 5 µM ascorbic acid (AA) were included in all experiments, and the results consistently showed that this concentration had no impact on cell viability. Data were normalized to the fluorescence measured in the 6-OHDA–treated group.

### 4.8. Cell Cycle Analysis

Cell cycle analysis was also performed to check for apoptotic cells by flow cytometry as previously described [42]. Briefly, after the treatments detailed before, the cells were fixed with 70% of ice-cold ethanol, treated with RNase (10 mg/mL), stained with PI (2 mg/mL), and incubated for 30 min at room temperature in darkness. Red fluorescence (DNA) was detected through a 563–607 nm band-pass filter using a BD FACSCalibur Flow Cytometry System (BD Bioscences, Milan, Italy). A minimum of 104 cells per sample were collected, and the percentage of apoptotic cells accumulated in the sub-G0/G1 peak was calculated by using Cell Quest Pro software version 3.3.

### 4.9. Animals

Male mice, inbred ICR—an inbred strain of mouse that is used as a general-purpose research strain and for therapeutic drug testing with a body weight of 20–30 g—were purchased from the National Breeding Center, Sofia, Bulgaria, and used for all experiments. All procedures were approved by the Bulgarian Food Safety Agency (Permission No. 323, valid until 22.12.2026) and were conducted in accordance with Ordinance No. 15/2006 for the humane treatment of experimental animals (vivarium certificate of registration No. 0072/01.08.2007). All experiments were performed and reported according to the ARRIVE guidelines (Animal Research: Reporting In Vivo Experiments) [45] and European Regulations for the Handling of Experimental Animals.

### 4.10. In Vivo Experimental Design

A PD model was developed in mice through the intraperitoneal administration of 3 mg/kg body weight of rotenone (Sigma-Aldrich, St. Louis, MO, USA) for 30 consecutive days. The animals were divided into 8 groups, with each group consisting of 6 animals [46].

### 4.11. Determination of GSH in Brain Homogenate

After precipitating the proteins with trichloroacetic acid, the thiol groups in the supernatant were determined using DTNB (5,5′-dithiobis (2-nitrobenzoic acid)), which produces a yellow-colored compound that absorbs light at λ = 412 nm. The brain homogenate was treated with 5% trichloroacetic acid and centrifuged at 6000× *g* for 5 min. The supernatant was then collected for GSH determination [47].

### 4.12. Determination of MDA in Brain Homogenate

The brain homogenate was precipitated with 25% trichloroacetic acid, followed by the addition of 0.67% thiobarbituric acid. This reaction results in the formation of a colored complex between the malondialdehyde and thiobarbituric acid. The determination of MDA was performed spectrophotometrically at 535 nm. A molar extinction coefficient of 1.56 × 10^5^ M^−1^ cm^−1^ was used for the calculations [48].

### 4.13. Histopathological Analysis

Samples from the mouse brains were fixed in 10% buffered neutral formalin solution. The fixed tissues were dehydrated in ascending grades of alcohol: 50%, 60%, 70%, 80%, 90%, 96%, and absolute alcohol. After dehydration, the samples were cleared in xylene and embedded in paraffin blocks. Sections with a thickness of 5 μm were prepared using a rotary microtome. The sections were attached to slides using histological adhesive and processed with xylene before being rehydrated in a descending alcohol series: absolute alcohol, 96%, 90%, 80%, 70%, 60%, and 50%. Finally, the sections were stained with Hematoxylin-Eosin [49]. Microscopic examination and photography were performed using a Levenhuk D740T light microscope with an integrated camera (Levenhuk, Tampa, FL, USA).

### 4.14. Statistical Analysis

The results from the in vivo experiments (GSH levels and MDA production) were statistically analyzed using the MEDCALC program and the non-parametric Mann–Whitney method, with significance levels set at *p* < 0.05, *p* < 0.01, and *p* < 0.001. For the statistical analyses involving the SH-SY5Y cell line and hMAO-A/B activity, GraphPad Prism software (version 8, GraphPad Software, La Jolla, CA, USA) was utilized. The significance of the data were assessed using one-way analysis of variance (ANOVA) followed by Dunnett’s test for post hoc multiple comparisons to evaluate statistical differences. Values of * *p* ≤ 0.05, ** *p* ≤ 0.01, and *** *p* ≤ 0.001 were considered statistically significant.

## 5. Conclusions

Overall, this study demonstrates that N-pyrrolyl hydrazide-hydrazone derivatives—particularly compound 2—exert robust neuroprotective effects both in vitro and in vivo, primarily through direct antioxidant effects, MAO-B inhibition, and antiapoptotic activity. Their selective efficacy in models of 6-OHDA-damaged cells, but not in those involving mitochondrial complex I inhibition by MPP+, suggests that these compounds may be most effective during stages of PD in which oxidative damage predominates over bioenergetic failure. Given their low cytotoxicity and significant efficacy across multiple assays, further development of these compounds is warranted. Future research should include comprehensive pharmacokinetic profiling and assessments of blood-brain barrier permeability, chronic toxicity evaluations, and behavioral testing in long-term PD models. Additionally, exploring combination therapies with agents targeting mitochondrial function may enhance therapeutic outcomes. Finally, structural optimization guided by structure-activity relationship (SAR) analysis and molecular docking studies could yield next-generation derivatives with broader neuroprotective spectra, potentially addressing both oxidative stress and mitochondrial dysfunction in PD.

## Figures and Tables

**Table 1:** IDs, chemical structures, and molecular weights of the target pyrrole-based hydrazide-hydrazones.

ID	ID in Reference	Chemical Structure	Molecular Weight g/mol	Reference
**1**	5a	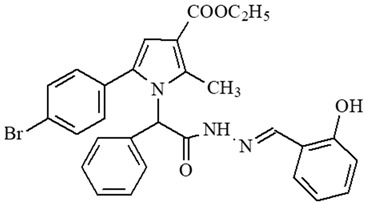	574.47	[11,25]
**2**	5o	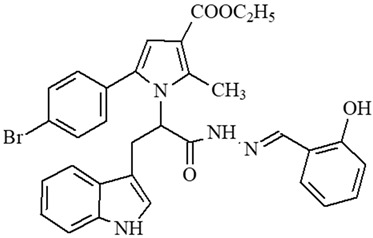	613.51	[26,29]
**3**	5j	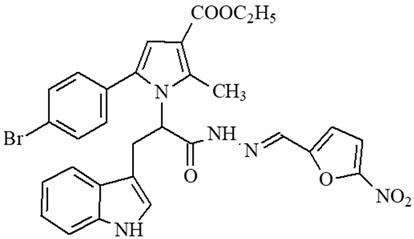	632.47	[26,29]
**4**	5a	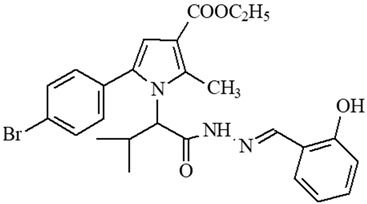	526.42	[24]
**5**	11	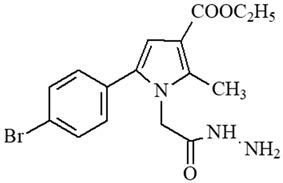	380.24	[15,22]
**6**	11b	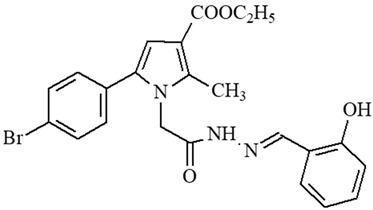	484.34	[15,22]
**7**	11l	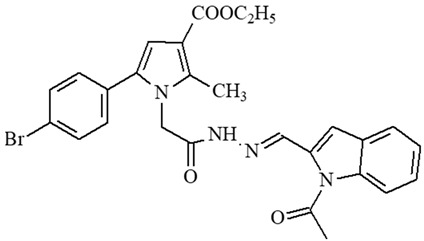	549.42	[15,22]
**8**	12	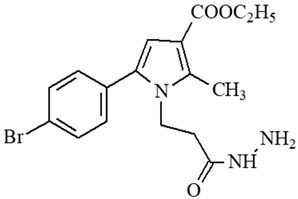	394.26	[15,23]
**9**	12a	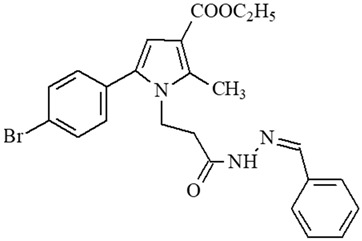	482.37	[15,23]
**10**	12b	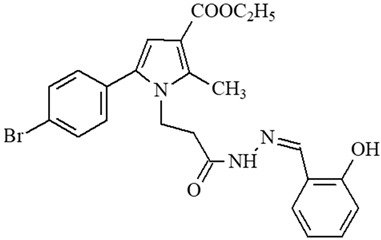	498.37	[15,23]

**Table 2:** Non-toxic concentrations of the test compound identified by in vitro cytotoxicity screening.

Compound		Compound	
**1**	1 μM	**6**	1 μM
**2**	1 μM	**7**	1 μM
**3**	1 μM	**8**	20 μM
**4**	1 μM	**9**	20 μM
**5**	1 μM	**10**	1 μM

**Table 3:** Experimental groups and treatment regimen in the rotenone-induced toxicity model.

Evaluation of Toxicity Following Repeated Dosing, i.p. for 30 Days		Model of Rotenone-Induced Toxicity, Rotenone + Compounds, i.p., for 30 Days	
Group 1—negative control	Saline 0.5 mL/g daily	Group 6	Rotenone 3 mg/kg + **2** 2 mg/kg daily
Group 2—positive control	Rotenone 3 mg/kg daily	Group 7	Rotenone 3 mg/kg + **4** 2 mg/kg daily
Group 3	**2** 2 mg/kg daily	Group 8	Rotenone 3 mg/kg + **5** 2 mg/kg daily
Group 4	**4** 2 mg/kg daily		
Group 6	**5** 2 mg/kg daily		

## Data Availability

The data is contained within the article.

## Supplementary Materials

The following supporting information can be downloaded at: https://www.mdpi.com/article/10.3390/ijms27010370/s1.

## Abbreviations

6-OHDA	6-hydroxydopamine
AA	Ascorbic acid
AD	Alzheimer’s disease
ALS	Amyotrophic lateral sclerosis
ATP	Adenosine triphosphate
BBB	Blood-brain barrier
COMT	Catechol-O-Methyl Transferase
DAT	Dopamine transporter
EDG	Electron-donating group
GSH	Reduced glutathione
HD	Huntington’ disease
hMAO-A	human Monoamine oxidase A
hMAO-B	human Monoamine oxidase B
MAO-A	Monoamine oxidase A
MAO-B	Monoamine oxidase B
MPTP	1-methyl-4-phenyl-1,2,3,6-tetrahydropyridine
MPP^+^	1-Methyl-1-PhenylPyridinium
MS	Multiple sclerosis
PI	Propidium iodide
PD	Parkinson’s disease
ROS	Reactive oxygen species
SNpc	Substantia nigra pars compacta
SAR	Structure-activity relationship
