# Supplementary material for: Neuroprotective and Antioxidant Activity of Newly Synthesized N-Pyrrolyl Hydrazide-Hydrazones in Experimental Models of Neurotoxicity In Vitro and In Vivo
Source: Int J Mol Sci. 2025 Dec 29;27(1):370. doi: 10.3390/ijms27010370 (PMC12785714; doi:10.3390/ijms27010370)
Supplement: Supplementary file 1 [file ijms-27-00370-s001.zip › ijms-4030239-supplementary.pdf]

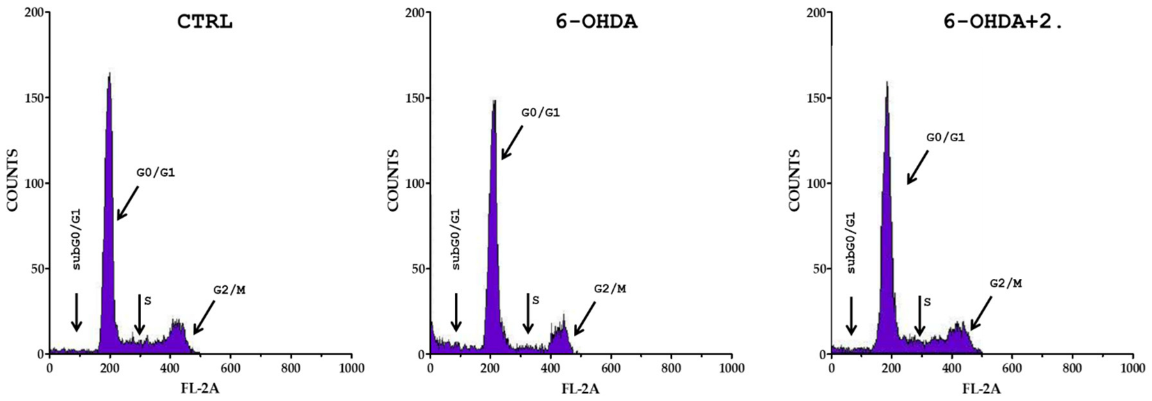

Figure S1. Exemplificative flow cytometric histograms of cell-cycle distribution in SH-SY5Y cells under three experimental conditions: untreated control (CTRL), 6-hydroxydopamine (6-OHDA) exposure, and 6-OHDA co-treatment with compound **2b**. DNA content (FL-2A) profiles display sub-G0/G1, G0/G1, S, and G2/M populations. 6-OHDA increases the sub-G0/G1 fraction, indicating apoptotic DNA fragmentation, while co-treatment with compound **2b** attenuates this effect and partially restores normal phase distribution, consistent with neuroprotection.
